# Radiographic Evaluation of Impacted and Transmigrant Canines: Prevalence and Sex-Based Differences in an Orthodontic Cohort

**DOI:** 10.3390/dj13090386

**Published:** 2025-08-26

**Authors:** Ioannis P. Zogakis, Chrysanthi Anagnostou, Ioulia Ioannidou, Stella Chaushu, Moschos A. Papadopoulos

**Affiliations:** 1 Department of Orthodontics, School of Dentistry, Aristotle University of Thessaloniki, 54124 Thessaloniki, Greece; 2 Department of Hygiene, Social-Preventive Medicine and Medical Statistics, School of Medicine, Aristotle University of Thessaloniki, 54124 Thessaloniki, Greece; 3 Department of Orthodontics, Hebrew University of Jerusalem, Hadassah Medical Center, Jerusalem 91120, Israel

**Keywords:** impacted canine(s), transmigration, prevalence, panoramic radiograph

## Abstract

**Background/Objectives**: Impacted teeth are characterized by having more than three-quarters of root development completed, however failing to erupt or demonstrate imminent eruption, as evidenced both by clinical and radiographic evaluation. Canine impaction is an entity of clinical significance considering its potential to affect both oral function and facial aesthetics. If not appropriately managed, this condition may adversely impact functional occlusion and structural smile integrity. The purpose of the study was to investigate the prevalence of impacted and transmigrant canines in a Greek orthodontic cohort and identify potential sex-based differences. **Methods:** A total of 2594 panoramic radiographs and clinical records of consecutive patients in the mixed and permanent dentition stages, treated at the Department of Orthodontics of the Aristotle University of Thessaloniki, Greece, were retrospectively retrieved and analyzed to detect the presence of impacted and transmigrant canines. Patients lacking complete clinical records or panoramic radiograph, undergoing or had undergone orthodontic treatment, or presenting conditions affecting normal permanent dentition development, including pathological conditions, cleft lip and palate or hereditary disorders, were excluded. **Results:** At least one impacted canine was detected in 109 patients (67% females, 33% males), with a median age of 15 years (IQR: 13–18), documenting a 4.2% prevalence (6.97% females, 2.32% males). The frequency of maxillary canine impaction was 3.97%, while in the mandible a frequency of 0.46% was reported. The frequency of bilateral canine impaction was 1%, with it being present in 25.7% of patients with canine impaction. The prevalence of transmigrant canines was 0.11%, with detection solely in the mandible. A statistically significant sex difference was detected solely in the prevalence of impaction. **Conclusions:** Considering the implications of canine impaction, the epidemiological investigation of this entity may facilitate an early diagnosis and treatment.

## 1. Introduction

Impacted teeth are defined as teeth having more than three-quarters of their root developed, not yet erupted or expected to erupt in the near future, as determined by the findings of the clinical and radiographical assessment [1]. The causative factor of impaction may be local or systemic [2]. Various factors have been suggested as responsible for the impaction of canines, such as the agenesis or anomaly of the adjacent lateral incisor, genetics, the presence of mechanical obstruction, inadequate space in the dental arch, prolonged retention of primary canine and syndromes such as cleidocranial dysplasia [1].

Another type of eruption disturbance is transmigration. Transmigration constitutes a rare entity and is defined as a pre-eruptive migration of a tooth from its normal site of development across the midline of the jaw [3]. A multitude of etiological factors have been proposed, including anomalous lateral incisors, hereditary factors, the abnormal position of the tooth germ, the retention or premature loss of deciduous teeth, trauma, dental crowding, the presence of supernumerary teeth or enlarged dental follicles and the excessive crown length of the mandibular canine. Localized pathological conditions such as tumors, cystic lesions and odontomata may also lead to transmigration of the teeth if they are positioned in the path of eruption [4,5].

Canine impaction is a phenomenon of clinical importance due to its functional and aesthetic implications to an individual. If left untreated, this condition may compromise both the functional occlusion and the smile architecture. It may also lead to the development of pathological conditions such as cystic lesions, root resorption of adjacent permanent teeth and consequently their exfoliation, thus compromising the dentition [1]. Therefore, a better understanding and an early diagnosis of impacted canines is crucial for orthodontic treatment planning.

Although numerous studies are available in the literature pertaining to the prevalence of canine impaction in different populations [6,7,8,9,10], to the authors’ best knowledge, there is no available data concerning the Greek population apart from some studies investigating the prevalence of teeth impaction in general, not thoroughly analyzing the characteristics of impacted canines or exploring possible sex-derived differences [11,12,13]. Therefore, the main purpose of the present study was to investigate the prevalence of canine impaction and transmigration in Greek orthodontic patients, while secondarily examining potentially existing sex-based differences.

## 2. Materials and Methods

The present study was designed as a retrospective observational study, and ethics approval for its conduction was obtained by the Bioethics Committee of the School of Dentistry of the Aristotle University of Thessaloniki, Greece (Nr. 25/21.11.2024). The subjects were patients who consecutively attended the Postgraduate Clinic of the Department of Orthodontics of the Aristotle University of Thessaloniki, Greece, whose clinical records included complete treatment history, with complete diagnostic and treatment notes and at least one pretreatment panoramic radiograph. Based on the protocol, a canine was classified as impacted when over 75% of its root development had occurred, yet it remained unerupted and was not anticipated to erupt in the foreseeable future. Cases in the mixed dentition stage were included provided that the permanent canines exhibited sufficient root development to allow for a reliable diagnosis of impaction. In instances involving severely ectopically positioned canines, the classification of impaction was applied even if less than three-quarters of the root had formed, based on the clinical judgment that spontaneous eruption was unlikely to occur due to the abnormal position of the tooth. The exclusion criteria for this study comprised patient records with incomplete clinical data or the absence of a panoramic radiograph prior to treatment initiation. Furthermore, individuals who had previously undergone or were currently undergoing orthodontic treatment at the time of data collection were deemed ineligible. In addition, patients presenting with any condition potentially affecting the normal development of permanent dentition, including pathological conditions, cleft lip and palate, or hereditary disorders and syndromes such as cleidocranial dysplasia, were excluded.

The presence and location of impacted canines were determined through radiological evaluation and examination of clinical notes documented on 2594 patients’ records. The radiographic evaluation was primarily based on panoramic radiographs, as CBCT imaging was not available for all patients. However, the diagnosis and determination of the buccolingual position of the canines were confirmed either through the examination of available CBCT scans, or via clinical notes documented during the surgical exposure, as recorded in the patients’ files. All of the patients’ pretreatment panoramic radiographs were viewed twice under standardized conditions by one experienced orthodontist (IPZ). Panoramic radiographs were viewed and evaluated by the examiner and a re-evaluation was conducted after a two-week interval. The intra-examiner reliability was assessed implementing Cohen’s Kappa. In the present study, Cohen’s Kappa = 0.995, *p* < 0.001; therefore, the intra-rater reliability was deemed as excellent.

The presence of transmigrated canines was also assessed, with impacted canines being considered as transmigrant when the canine’s cusp tip had crossed the skeletal midline. Transmigrant canines were classified in five types based on their migratory pattern and the final position in the jaw, according to Mupparapu’s classification [3]: Type 1: The impacted canine is positioned mesio-angularly across the midline, labially or lingually related to the anterior teeth, with the canine crown crossing the midline; Type 2: The canine is horizontally impacted in vicinity to the inferior border of the mandible below the incisors’ apices; Type 3: The canine is erupting mesial or distal to the canine of the opposite side; Type 4: The canine is horizontally impacted in vicinity to the inferior border of the mandible below the apices of premolars or molars on the opposite side; Type 5: The canine is positioned vertically in the midline, with the long axis of the tooth crossing the midline, irrespective of its eruption. All the radiographs of patients with transmigrant canines were viewed and traced on acetate tracing paper with a 0.3 mm-diameter lead pencil by the main investigator (IPZ).

Data were collected and analyzed using the open Jamovi software (Version 2.3, The Jamovi project (2024), Sydney, Australia). The Shapiro–Wilk test examined the distribution of continuous variables. Categorical variables are summarized by absolute and relative frequencies. Chi-squared tests were employed to compare categorical, qualitative, independent variables. Significance level was set at 0.05, two-tailed (*p* < 0.05).

## 3. Results

Treatment records of 2594 consecutive patients (59.7% males, 40.3% females) seeking orthodontic treatment were included in this study. In total, 109 patients (males = 36, females = 73) with 143 impacted canines were detected, documenting a 4.2% prevalence (6.97% in females, 2.32% in males). They had a median age of 15 years (IQR: 13–18) and the majority of them were females (67%). Statistically significant difference of canine impaction was detected between males and females (χ2 = 33.6, *p* < 0.001), with a ratio of 1:3 (Table 1).

The frequency of bilateral canine impaction was calculated at 1%, with it being present in 25.7% of patients with canine impaction. A higher frequency of unilateral (74.3%) over bilateral (25.7%) impactions was documented. Although more bilateral impactions were reported in female subjects (n = 19), as opposed to male (n = 9), the difference was not statistically significant (*p* = 0.9). A summary of the documented frequencies of canine impaction is demonstrated in Table 2.

The frequency of maxillary canine impaction was 3.97%, while mandibular impacted canines demonstrated a frequency of 0.46%. The majority of impacted canines were located in the maxilla (n = 130, 90.9%), with the remaining 9.1% (n = 13) being located in the mandible. The majority of canines (n = 76, 53.1%) were positioned palatally, with the remaining 46.9% being positioned buccally. No statistically significant sex differences were detected concerning the jaw (*p* = 0.11), the buccolingual position (*p* = 0.74), or the side (*p* = 0.64) of impaction.

Regarding the maxillary impacted canines, 76 of them were located palatally (58.5%) as opposed to 41.5% being impacted buccally (n = 54), documenting a palatal-to-buccal ratio of 1.5 to 1. The greatest part of them (58.5%, n = 76) were impacted on the right quadrant. Although 68.9% of palatally impacted canines were documented in females, no statistical significance was identified between males and females (*p* = 0.98). All of the mandibular canines (n = 13) were positioned buccally with seven (53.8%) of them being positioned on the left quadrant. The summary of the impacted canines’ characteristics is demonstrated in Table 3.

In addition, three of the 2594 examined radiographs, in three different patients (two of them being males), exhibited three impacted transmigrant canines, documenting a prevalence of 0.11%. According to Mupparapu’s classification [3], one impacted canine was Type 1, while the other two were Type 2. All of the transmigrant canines were buccally impacted, with two of them being left-sided (66.7%). No transmigrant maxillary canines were detected in this study.

## 4. Discussion

Canine impaction constitutes a clinical entity that may affect both the functional occlusion and smile aesthetics. The prevalence of canine impaction varies depending on the population studied; therefore, it is deemed imperative to have available data for as many population groups as possible. The present study documented a 4.2% frequency of the presence of at least one impacted canine in Greek orthodontic patients. The frequency of maxillary canine impaction was calculated at 3.97%, similar to numerous studies reporting it with a range from 0.9% to 5%, depending on the population [2,14,15,16,17]. Mandibular canine impaction was more infrequent in Greek patients, with a reported frequency of 0.46%, which was in accordance with previous studies, reporting a prevalence ranging from 0.3–5.1% [2,9,17] and with two studies concerning Greek patients documenting a 0.4% and a 0.5% incidence [11,13]. Canine impaction may implicate one side (unilateral), or both (bilateral). This study documented a 1% frequency of bilateral canine impaction in Greek orthodontic patients, with it being present in 25.7% of patients with canine impaction, thus highlighting a higher frequency of unilateral over bilateral impactions [10,12].

In light of sex distribution, solely the presence of at least one impacted canine was found significantly higher in females when compared to males, with a female-to-male ratio of 3:1, a finding coming in accordance with other studies reporting higher frequency in female patients [8,11,18,19], as well as ratios of 2.4:1 in a previous study conducted in Greek patients [12] and 3:1 in orthodontic patients in India [10]. However, an equal sex distribution has also been reported [9]. Moreover, similar to Brin et al.’ s findings, our study could not detect a significant female predominance of palatal canine impaction [20].

Regarding the position of impaction, a palatal-to-buccal ratio of 1.5 to 1 was documented for maxillary impaction, a finding in accordance with various studies supporting the maxillary impacted canine being more often located palatally than labially [8,11,14,15,21,22], while all the mandibular canines were impacted buccally. Comparison of the canine impaction between the right and the left side concluded a higher frequency of right-side impactions, as similarly reported in the literature [10], although a left-side impaction predominance has been also documented [9].

Mandibular canine impaction, and particularly transmigration, are infrequent [2]. According to numerous studies, the prevalence of canine transmigration ranges from 0.004–0.98% [6,10,23,24,25,26,27]. The present study reported a prevalence of 0.11% of transmigrant canines in Greek orthodontic patients. As opposed to findings of various studies, including one concerning Greek patients, reporting cases of maxillary transmigrant canines [7,23,25], our study identified transmigrant canines solely in the mandible.

### Strengths and Limitations

Although a considerable body of literature addresses the prevalence of canine impaction across various populations, there appears to be a lack of comprehensive data specific to the Greek population. To the best of the authors’ knowledge, the existing Greek studies primarily focus on the overall prevalence of dental impactions, without providing a detailed analysis of the clinical and radiographic characteristics of impacted canines or examining potential differences related to sex. Therefore, the present investigation represents the first study to specifically address this issue within the Greek population.

The results of the current investigation may be in accordance with the findings of numerous other studies; however, one may detect variations that can be attributed not only to dissimilarities in the sample size and selection and the regional characteristics of the participants, but also to the racial, genetic and ethnic dissimilarities, considering that this type of study is population-oriented. Therefore, should we aim to draw reliable conclusions that could assist the clinician in conducting an early diagnosis of this entity, we should acquire data pertaining to the specific population of interest, rendering data collection for as many population groups as possible imperative for the conduction of future studies. An additional limitation of this study arises from the use of panoramic radiographs, being two-dimensional imaging modalities, as the primary diagnostic tool. However, as abovementioned, the diagnosis and buccolingual positioning of each impacted canine have been confirmed either through the evaluation of available CBCT scans, or based on clinical documentation recorded during surgical exposure, as detailed in the patients’ files. Another limitation that should be taken into account is that the sample comprised patients seeking orthodontic treatment rather than individuals from a general community population, which may have resulted in a slight overestimation of prevalence rates.

Awareness of the prevalence rates of canine impaction and transmigration, particularly in relation to other patient-specific characteristics, within the target population, may aid clinicians in the early detection of this clinical entity. Early recognition of impacted canines could enable timely intervention, mitigating harmful effects and related conditions, such as the development of cystic formations accompanied by resorptive procedures affecting neighboring permanent dentition, thereby minimizing their potential functional and aesthetic consequences.

## 5. Future Directions

As stated above, greater emphasis should be placed on population-based research into the prevalence and patterns of canine impaction and transmigration, particularly in association with patient-specific factors. Such investigations hold potential to enhance clinical awareness and enable earlier identification of at-risk individuals. Timely diagnosis is essential for initiating appropriate interventions that can prevent or limit complications including functional or aesthetic impairments. By refining diagnostic protocols and integrating prevalence data into clinical practice, healthcare professionals may improve treatment outcomes and reduce the long-term burden associated with undiagnosed or late-detected cases of impacted canines.

## 6. Conclusions

In the present study, the prevalence of canine impaction in Greek orthodontic patients was 4.2%, significantly higher in females (6.97%) than in males (2.32%). No other statistically significant sex-based difference could be identified, when examining for bilateral impaction, jaw, buccolingual position, side of impaction and frequency of palatally impacted canines. The frequency of maxillary impacted canines was 3.97%, while mandibular canine impaction was scarce, with a frequency of 0.46%. Transmigrant canines were detected solely in the mandible, with a prevalence of 0.11%. In light of the significant functional and aesthetic implications associated with canine impaction, an elaborate, population-oriented investigation of this phenomenon holds considerable clinical value. Such research may not only enhance the accuracy and timeliness of diagnosis but also enable the implementation of targeted preventive and therapeutic interventions, thereby mitigating potential adverse outcomes and reducing the risk of associated pathological conditions.

## Figures and Tables

**Table 1:** Association of the presence of at least one impacted canine and sex.

Sex	At Least One Impacted Canine		
	Yes	No	Total
Male	36 (2.3%)	1512 (97.7%)	1548 (100%)
Female	73 (7%)	973 (93%)	1046 (100%)
Total	109 (4.2%)	2485 (95.8%)	2594 (100%)

*p* < 0.001.

**Table 2:** Summary of frequencies of impaction.

	Patients	≥1 Impacted Canine	Bilateral Impaction	Impaction in Both Jaws	3 Impacted Canines
**Sex**					
Male	1548 (59.7%)	36 (33%)	9 (32.1%)	2 (33.3%)	2 (66.7%)
Female	1046 (40.3%)	73 (67%)	19 (67.9%)	4 (66.7%)	1 (33.3%)
Total	2594 (100%)	109 (100%)	28 (100%)	6 (100%)	3 (100%)
**Frequency**		4.2%	1%	0.23%	0.11%

**Table 3:** Summary of impacted canines’ characteristics.

	Jaw		
Characteristics	Maxilla	Mandible	Total
**Side**			
Right	76 (58.5%)	6 (46.2%)	82 (57.3%)
Left	54 (41.5%)	7 (53.8%)	61 (42.7%)
**Position**			
Palatal	76 (58.5%)	0 (0%)	76 (53.1%)
Buccal	54 (41.5%)	13 (100%)	67 (46.9%)
**Total**	130 (90.9%)	13 (9.1%)	143 (100%)

## Data Availability

The datasets generated during and/or analyzed during the current study are available from the corresponding author upon reasonable request.
